# Digital intervention (Renewed) to support symptom management, wellbeing, and quality of life among cancer survivors in primary care: a randomised controlled trial

**DOI:** 10.3399/BJGP.2023.0262

**Published:** 2025-01-14

**Authors:** Paul Little, Katherine Bradbury, Beth Stuart, Jane Barnett, Adele Krusche, Mary Steele, Elena Heber, Steph Easton, Kirsten A Smith, Joanna Slodowska-Barabasz, Liz Payne, Teresa Corbett, Laura Wilde, Guiqing Lily Yao, Sebastien Pollet, Jazzine Smith, Judith Joseph, Megan Lawrence, Dankmar Böhning, Tara Cheetham-Blake, Diana Eccles, Claire Foster, Adam WA Geraghty, Geraldine Leydon, Andre Matthias Müller, Richard D Neal, Richard Osborne, Shanaya Rathod, Alison Richardson, Chloe Grimmett, Geoffrey Sharman, Roger Bacon, Lesley Turner, Richard Stephens, Kirsty Rogers, James Raftery, Shihua Zhu, Karmpaul Singh, Frances Webley, Gareth Griffiths, Jaqui Nutall, Trudie Chalder, Clare Wilkinson, Eila Watson, Lucy Yardley

**Affiliations:** Primary Care Research Centre, University of Southampton, Southampton, UK.; Department of Psychology, University of Southampton, Southampton, UK.; Primary Care Research Centre, University of Southampton, Southampton, UK.; Primary Care Research Centre, University of Southampton, Southampton, UK.; Department of Psychology, University of Southampton, Southampton, UK.; Department of Psychology, University of Southampton, Southampton, UK.; Department of Psychology, University of Southampton, Southampton, UK.; Department of Psychology, University of Southampton, Southampton, UK.; Primary Care Research Centre, University of Southampton, Southampton, UK.; Oxford Institute of Nursing, Midwifery and Allied Health Research, Oxford Brookes University, Oxford, UK.; Department of Psychology, University of Southampton, Southampton, UK.; School of Health Sciences, University of Southampton, Southampton, UK.; Faculty of Health & Life Sciences, Coventry University, Coventry, UK.; Biostatistics Research Group, University of Leicester, Leicester, UK.; Department of Psychology, University of Southampton, Southampton, UK.; Department of Psychology, University of Southampton, Southampton, UK.; Department of Psychology, University of Southampton, Southampton, UK.; Southampton Clinical Trials Unit, University of Southampton, Southampton, UK.; Mathematical Sciences, University of Southampton, Southampton, UK.; School of Health Sciences, University of Southampton, Southampton, UK.; Southampton Clinical Trials Unit, University of Southampton, Southampton, UK.; Centre for Psychosocial Research in Cancer: CentRIC+, Health Sciences, University of Southampton, Southampton, UK.; Primary Care Research Centre, University of Southampton, Southampton, UK.; Primary Care Research Centre, University of Southampton, Southampton, UK.; Saw Swee Hock Public School of Health, National University of Singapore, Singapore.; College for Medicine and Health, University of Exeter, Exeter, UK.; Dorset Cancer Centre, Poole, UK.; Southern Health NHS Foundation Trust, Southampton, UK.; School of Health Sciences, University of Southampton, Southampton, and University Hospital Southampton NHS Foundation Trust, Southampton, UK.; School of Health Sciences, University of Southampton, Southampton, UK.; Health Economics Analysis Team, University of Southampton, Southampton, UK.; Health Economics Analysis Team, University of Southampton, Southampton, UK.; Health Economics Analysis Team, University of Southampton, Southampton, UK.; Primary Care Research Centre, University of Southampton, Southampton, UK.; Primary Care Research Centre, University of Southampton, Southampton, UK.; Health Economics Analysis Team, University of Southampton, Southampton, UK.; Health Economics Analysis Team, University of Southampton, Southampton, UK.; Professor, Department of Psychology, University of Calgary, Calgary, Canada.; Southampton Clinical Trials Unit, University of Southampton, Southampton, UK.; Southampton Clinical Trials Unit, University of Southampton, Southampton, UK.; Southampton Clinical Trials Unit, University of Southampton, Southampton, UK.; Department of Psychological Medicine, Institute of Psychiatry, Psychology and Neuroscience, King’s College London, London, UK.; School of Health Sciences, Bangor University, Bangor, UK.; Oxford Institute of Nursing, Midwifery and Allied Health Research, Oxford Brookes University, Oxford, UK.; Department of Psychology, University of Southampton, Southampton, and School of Psychological Science, University of Bristol, Bristol, UK.

**Keywords:** cancer survivors, health resources, primary health care, self-management

## Abstract

**Background:**

Many cancer survivors following primary treatment have prolonged poor quality of life.

**Aim:**

To determine the effectiveness of a bespoke digital intervention to support cancer survivors.

**Design and setting:**

This was a pragmatic parallel open randomised trial in UK general practices (ISRCTN:96374224).

**Method:**

People having finished primary treatment (≤10 years previously) for colorectal, breast, or prostate cancers, with European Organization for Research and Treatment of Cancer Core Quality of Life Questionnaire (EORTC QLQ-C30) score ≤85, were randomised by online software to: 1) detailed ‘generic’ digital NHS support (‘LiveWell’; *n* = 906); 2) a bespoke complex digital intervention (‘Renewed’; *n* = 903) addressing symptom management, physical activity, diet, weight loss, and distress; or 3) ‘Renewed with support’ (*n* = 903): ‘Renewed’ with additional brief email and telephone support.

**Results:**

Mixed linear regression provided estimates of the differences between each intervention group and generic advice. At 6 months all groups improved (primary time point: *n* for the generic, Renewed groups, and Renewed with support were 806, 749, and 705, respectively), with no significant between-group differences for EORTC QLQ-C30, but global health improved more in both the Renewed groups. By 12 months there were small improvements in EORTC QLQ-C30 for Renewed with support (versus generic advice: 1.42, 95% confidence interval [CI] = 0.33 to 2.51); both Renewed groups improved global health (12 months: Renewed: 3.06, 95% CI = 1.39 to 4.74; Renewed with support: 2.78, 95% CI = 1.08 to 4.48), dyspnoea, constipation and enablement, and lower primary care NHS costs (in comparison with generic advice [£265]: Renewed was –£141 [95% CI = –£153 to–£128] and Renewed with Support was –£77 [95% CI = –£90 to –£65]); and for Renewed with support improvement in several other symptom subscales. No harms were identified.

**Conclusion:**

Cancer survivors’ quality of life improved with detailed generic online support. Robustly developed bespoke digital support provides limited additional short-term benefit, but additional longer-term improvement in global health, enablement, and symptom management, with substantially lower NHS costs.

## Introduction

Although the UK has one of the poorest cancer survival rates among higher-income settings,^1^ the prevalence of cancer survivors has been increasing year on year (https://www.cancerresearchuk.org) and by 2040 cancer survivors are likely to represent a quarter of the UK population.^2^ The quality of life (QoL) of some cancer survivors is poor, equivalent to chronic diseases^3^^,^^4^ and remains persistently poor over years.^5^^,^^6^

Existing interventions to improve QoL are usually delivered by healthcare practitioners.^7^ It can be difficult to roll out clinician-based complex behaviour change interventions at scale, because in practice clinicians often lack the time or behavioural counselling skills needed to provide such support.^8^ Digital interventions may help,^9^ cancer survivors perceive them positively,^9^ and some can be effective^9^^–^11 — albeit with limited tailoring, mostly small trials, and few in typical primary care settings. In the UK, there is limited evidence for digital interventions designed for stable cancer survivors to promote cognitive and behavioural changes to improve overall QoL and health. The current author group developed a digital intervention (‘Renewed’) using the person-based-approach,^12^ co-producing the intervention with cancer survivors and clinical experts, optimised based on feedback on prototypes.^13^

**Table:** How this fits in

The number of cancer survivors is increasing, many whose quality of life remains consistently poor over years. There is limited robust evidence for pragmatic, brief interventions to support cancer survivors in primary care — which is where most participants are managed, and where resources are increasingly stretched. Cancer survivors’ quality of life improved with detailed generic online support. Robustly developed bespoke digital support provided limited additional benefit for cancer survivors in the short term, but modest additional longer-term benefit in enabling symptom management and self-rated health, and with significantly reduced costs to the health service.

The main results from the randomised controlled trial of Renewed are reported in the current paper. The aim was to assess whether the Renewed intervention, with or without human support, resulted in a difference in QoL and overall wellbeing compared with access to detailed generic advice.

## Method

The protocol for the main trial has been published.^14^

### Setting and participants

The trial was conducted in NHS general practices; all but one were from England and Wales (one in Scotland). The study was registered on the ISRCTN database on 9 August 2017 (ISRCTN 96374224) before the recruitment of the first participant.

#### Participant inclusion criteria

Contrasting groups of participants (breast cancer survivors [younger and older females]; prostate cancer survivors [predominantly older males]; and colorectal cancer [a range of ages and gender]) were identified from case record searches. They had completed primary treatment (≥1 month and ≤10 years previously), had internet access, and scored ≤85 on the European Organization for Research and Treatment of Cancer Core Quality of Life Questionnaire (EORTC QLQ-30, the lowest-scoring two-thirds of the patient group^6^). Participants were mainly recruited by invitation but could be recruited opportunistically.

#### Participant exclusion criteria

Palliative care, active cancer (unless prostate cancer active watchful waiting), another type of cancer in the past 5 years, current or expected cancer treatment (except hormones), severe mental health problems, breast sarcoma/lymphoma, and in the same household as another participant were exclusion criteria.

#### Randomisation

Automated randomisation with a 1:1:1 allocation ratio using LifeGuide software (www.lifeguideonline.org), stratified by:

- cancer type: breast/prostate/colorectal; and
- EORTC QLQ-C30 score (≤64/≥65 — the cut-off for the lowest 25% of the distribution^6^^,^^15^).

### Interventions

The development of ‘Renewed’ has been published.^13^ Detail of the content is given in Supplementary Information S1. The trial groups were as follows:

- detailed ‘generic’ digital NHS support (‘Live Well’; *n* = 906);
- a bespoke complex digital intervention (‘Renewed’; *n* = 903) addressing symptom management, physical activity, diet, weight loss, and distress; or
- ‘Renewed with support’ (*n* = 903): ‘Renewed’ with additional brief support by email and telephone.

### Measures and outcomes

Outcomes were patient self-reported online (baseline, 6, and 12 months unless indicated), and for non-responders two email reminders, two postal administrations (and a £10 voucher at 6 months for all responders), and a final telephone follow-up made blind to group. Information from medical records was obtained blind to group.

#### Primary outcome

The primary outcome was QoL measured using the EORTC QLC-30 instrument (version 3) summary score.^16^

#### Secondary outcomes

Secondary outcomes were:

- EORTC QLQ-C30 subscales (baseline, 6, and 12 months): global self-rated health, symptom subscales, functional subscales (for example, physical functioning, social functioning, emotional wellbeing);
- depression and anxiety,^17^ fear of relapse,^18^ and the Measure Yourself Concerns and Wellbeing questionnaire (MYCaW) for QoL^19^ (baseline, 12 months);
- modified enablement scale^20^^,^^21^ (12 months);
- resource use (medication/consultation costs in primary care);
- other outcomes/measures will be reported in the process analysis in a separate article (a website satisfaction measure [12 months]; Problematic experiences of therapy scale (PETS) for self-reported adherence); and
- website usage.

### Sample size

It was estimated that to detect a 0.3 standardised mean difference (SMD) between the intervention and control for each cancer type (80% power; alpha = 0.05) required 176 intervention and 176 control participants, 1584 for the three cancers, or 1980 allowing for 20% loss to follow-up. It was estimated that the total sample would detect overall differences between intervention groups of 0.15 SMDs, which is more realistic for a brief intervention. Cluster effects are possible even in individually randomised designs: assuming eight participants per intervention group per practice, an intraclass correlation coefficient of 0.03 (inflation factor 1.21: 1 + [8 – 1 × 0.03]), required 2396 participants. Allowing for some leeway, the aim was to recruit 2500 individuals.

### Statistical methods

In accordance with the ISRCTN registration, following a ‘feasibility’ phase, the initial feasibility study became an internal pilot, providing data for analysis of the whole trial. Data were analysed on an intention-to-treat basis using Stata version 17 (Statacorp), but the 20 participants were excluded post randomisation largely for reasons of ineligibility (Supplementary Figure S1). A statistical analysis plan is available on request from the corresponding author. Generalised linear mixed regression models were used for the analysis of continuous variables, controlling for baseline values, stratification variables, covariates (in accordance with International Council for Harmonisation of Technical Requirements for Pharmaceuticals for Human Use E9 guidance), and a random effect (a random intercept) for practice. The primary analysis used a chained equation multiple imputation model for missing data including all outcomes (including 6- and 12-month time points) as well as all variables included in the analysis model. A chained-equations approach was used with a distribution suitable to each variable. This was linear for EORTC QLQ-C30, Hospital Anxiety and Depression Scale (HADS), MYCaW, enablement, and body mass index. A logistic model was used for the presence/absence of comorbidities. Guidance suggests that the number of imputations should equal or exceed the percentage of missing data,^22^ which would have been 25 in this case, but to minimise the bias in estimating the point estimates, confidence intervals (CIs) and *P*-values 100 was chosen in the current study. A complete cases analysis was a sensitivity analysis.

### NHS resource use

Resource use data were collected by a medical record review in primary care — and primary care was the primary focus in this study, as that was where it was anticipated any differences in resource use might be found (Supplementary Information S2).

## Results

### Recruitment

A total of 58 295 ‘cold calling’ invitations were sent by mail from 494 GP practices and 7883 individuals expressed interest (see Supplementary Table S1 for reasons for non-participation). In total, 2732 participants were recruited between 12 October 2017 and 2 April 2020, and the most common reason for not wanting to participate was not having current problems (Supplementary Figure S1 and Supplementary Table S1); 20 patients were excluded post-randomisation largely because of being ineligible (for example, living at the same address).

### Engagement

Patients accessed Renewed a median of two times (range 0–268). Most (96.8%, 1703/1760) accessed the core content of Renewed, with 84.4% (1487/1760) completing the core content and reaching the ‘Homepage’ for optional content suitable for their particular context.^9^ The optional contents of Renewed were accessed by 45.0% (790/1760). Of those offered facilitator support, 31.1% (235/756) chose to access it.

### Baseline characteristics

These were well balanced between groups (Table 1 and Table 2) and between cancers (Supplementary Table S2) except for gender differences between prostate and breast.

**Table 1.** Baseline characteristics by intervention group

**Characteristics**	**Generic advice (*n* = 906)**	**Renewed with support (*n* = 903)**	**Renewed (*n* = 903)**	**Total (*n* = 2712)**
**Age, years, mean (SD)**	64.5 (10.9)	64.5 (11.2)	64.5 (10.7)	64.5 (10.9)

**Baseline EORTC QLQ-C30 score,^a^** **mean (SD)**	72.1 (12.2)	72.5 (11.8)	72.7 (11.7)	72.4 (11.9)

**Education status**				
School leaver	397/906 (43.8)	412/903 (45.6)	377/903 (41.7)	1186/2712 (43.7)
College	231/906 (25.5)	237/903 (26.2)	241/903 (26.7)	709/2712 (26.1)
Degree or higher	278/906 (30.7)	254/903 (28.1)	285/903 (31.6)	817/2712 (30.1)

**Marital status**				
Single	64/906 (7.1)	46/902 (5.1)	53/902 (5.9)	163/2710 (6.0)
Living with partner	52/906 (5.7)	73/902 (8.1)	63/902 (7.0)	188/2710 (6.9)
Married	646/906 (71.3)	624/902 (69.2)	639/902 (70.8)	1909/2710 (70.4)
Divorced	65/906 (7.2)	82/902 (9.1)	76/902 (8.4)	223/2710 (8.2)
Widowed	58/906 (6.4)	61/902 (6.8)	59/902 (6.5)	178/2710 (6.6)
Separated	21/906 (2.3)	16/902 (1.8)	12/902 (1.3)	49/2710 (1.8)

**Ethnicity**				
Ethnic minority	22/906 (2.4)	19/902 (2.1)	20/902 (2.2)	61/2710 (2.3)
White	884/906 (97.6)	883/902 (97.9)	882/902 (97.8)	2649/2710 (97.8)

**Body mass index, mean (SD)**	28.0 (5.5)	28. 2 (5.5)	28.0 (5.4)	28.1 (5.5)

**Cancer group**				
Bowel/colorectal	143/906 (15.8)	143/903 (15.8)	146/903 (16.2)	432/2712 (15.9)
Breast	474/906 (52.3)	471/903 (52.2)	471/903 (52.2)	1416/2712 (52.2)
Prostate	289/906 (31.9)	289/903 (32.0)	286/903 (31.7)	864/2712 (31.9)

**Comorbidities^b^**				
Cardiovascular	291/779 (37.4)	325/787 (41.3)	329/789 (41.7)	945/2355 (40.1)
Lung	130/779 (16.7)	126/787 (16.0)	149/789 (18.9)	405/2355 (17.2)
Other	548/779 (70.3)	545/787 (69.3)	563/789 (71.4)	1656/2355 (70.3)

**Gender**				
Male	379/906 (41.8)	380/903 (42.1)	368/903 (40.8)	1127/2712 (41.6)
Female	527/906 (58.2)	523/903 (57.9)	535/903 (59.2)	1585/2712 (58.4)

**Time since last cancer treatment,** **years, mean (SD)**	4.0 (3.0)	4.1 (3.2)	3.9 (3.3)	4.0 (3.1)

*Data are* n/N *(%) unless otherwise indicated.*

a *Range 0–100, higher scores reflect higher quality of life.*

b *Participants could have more than one comorbidity. EORTC QLQ-C30 = European Organization for Research and Treatment of Cancer Core Quality of Life Questionnaire. SD = standard deviation.*

**Table 2.** Baseline EORTC QLQ-C30 subscale by intervention group

**Subscales**	**Mean (SD)**			

	**Generic advice (*n* = 906)**	**Renewed with support (*n* = 903)**	**Renewed (*n* = 903)**	**Total (*n* = 2712)**
**Global health^a^**	60.4 (17.3)	60.7 (17.0)	59.9 (16.2)	60.3 (16.8)

**Functional subscales^a^**				
Physical function	76.9 (19.0)	75.7 (19.4)	76.9 (17.8)	76.5 (18.7)
Role function	66.9 (26.6)	68.0 (26.1)	67.6 (25.6)	67.5 (26.1)
Emotional function	64.4 (22.6)	64.2 (23.2)	65.2 (22.6)	64.6 (22.8)
Cognitive function	69.9 (22.1)	71.2 (21.4)	72.4 (20.8)	71.2 (21.4)
Social function	66.1 (26.0)	67.1 (26.6)	66.9 (25.7)	66.7 (26.1)

**Symptom subscales^b^**				
Fatigue	44.7 (20.3)	44.2 (20.6)	44.3 (19.4)	44.4 (20.1)
Nausea and vomiting	6.9 (13.2)	6.8 (12.7)	7.0 (12.6)	6.9 (12.8)
Pain	33.4 (27.1)	32.8 (26.8)	32.6 (27.6)	32.9 (27.1)
Dyspnoea	24.1 (26.5)	23.2 (25.9)	24.2 (26.5)	23.8 (26.3)
Insomnia	50.8 (31.9)	50.6 (31.3)	50.4 (30.3)	50.6 (31.2)
Appetite loss	14.0 (22.3)	13.4 (21.2)	12.8 (21.3)	13.4 (21.6)
Constipation	19.9 (26.5)	20.0 (26.3)	18.6 (27.2)	19.5 (26.7)
Diarrhoea	13.7 (22.4)	13.0 (22.4)	14.5 (23.8)	13.8 (22.9)
Financial difficulties	11.6 (24.3)	12.8 (24.4)	11.8 (24.3)	12.1 (24.3)

a *Range 0–100, higher scores reflect improved health or functioning.*

b *Range 0–100, lower scores reflect improved symptom control. EORTC QLQ-C30 = European Organization for Research and Treatment of Cancer Core Quality of Life Questionnaire. SD = standard deviation.*

### Follow-up

By both 6 months 83.3% (2260/2712) and by 12 months 82.9% (2247/2712) had complete primary outcome data, with slight differences between group: 103/906 for generic advice care (11.4%), 201/903 (22.3%) for Renewed with support, and 221/903 (24.5%) for Renewed by 6 months. The follow-up rate for resource use was 86.7% (2351/2712).

### EORTC QLQ-30

There was improvement in all groups at 6 months (Tables 2 and 3, Supplementary Figure S2 and Supplementary Table S3), with no significant between-group differences. However, the Renewed with support group had higher QoL at 6 months than the generic advice group in the prostate cancer subgroup (2.03, 95% CI = 0.25 to 3.80). By 12 months there were small improvements in the EORTC QLQ-C30 for Renewed with support (1.42, 95% CI = 0.33 to 2.51). All cancer groups improved but the results were only significant for prostate cancer (Supplement Table S3).

**Table 3.** EORTC QLQ-30 at 6 and 12 months: means for each group, and adjusted differences between Renewed groups and generic advice

**EORTC QLQ-C30 scores**	**All participants**		

	**Generic advice**	**Renewed with support**	**Renewed**
**6 months**			
Score, mean (SD)	76.0 (14.31)	76.7 (14.41)	76.1 (13.99)
Complete cases, *n*	806	705	749
Difference in means (95% CI)			
Complete cases	Reference	0.50 (−0.67 to 1.66)	−0.42 (−1.57 to 0.72)
Imputed (100 imputations)	Reference	0.52 (−0.53 to 1.57)	−0.20 (−1.23 to 0.84)

**12 months**			
Score, mean (SD)	75.7 (15.13)	77.2 (14.07)	77.0 (14.42)
Complete cases, n	803	702	742
Difference in means (95% CI)			
Complete cases	Reference	1.11 (−0.10 to 2.31)	0.72 (−0.46 to 1.91)
Imputed (100 imputations)	Reference	**1.42 (0.33 to 2.51)^a^**	0.94 (−0.13 to 2.01)

a *Bold = significant at the 5% level. CI = confidence interval. EORTC QLQ-C30 = European Organization for Research and Treatment of Cancer Core Quality of Life Questionnaire. SD = standard deviation.*

### Results for EORTC QLQ-30 subscales at 6 months

There were significant differences compared with generic advice for self-rated global health in both Renewed groups (Table 4). The Renewed with support group also showed improvement in the physical function and cognitive function subscales.

**Table 4.** Results for EORTC QLC-30 subscales at 6 months: means for each group, and adjusted differences between Renewed groups and generic advice

**Subscales**	**Generic advice**	**Renewed with support**		**Renewed**	

	**Mean (SD)**	**Mean (SD)**	**Imputed (100 imputations) (95% CI)**	**Mean (SD)**	**Imputed (100 imputations)**
**Global health^a^**	64.4 (19.89)	66.3 (18.54)	**1.82 (0.14 to 3.52)^b^**	65.9 (19.11)	**1.88 (0.18 to 3.58)**

**Functional subscales^c^**					
Physical function	77.7 (20.68)	78.7 (19.88)	**2.00 (0.64 to 3.36)**	78.2 (19.88)	0.50 (−0.82 to 1.82)
Role function	71.9 (28.36)	71.3 (28.14)	−1.01 (−3.45 to 1.42)	71.0 (27.49)	−1.06 (−3.44 to 1.31)
Emotional function	69.8 (22.72)	70.5 (22.15)	0.79 (−1.01 to 2.60)	70.0 (21.93)	−0.05 (−1.87 to 1.76)
Cognitive function	73.5 (22.10)	76.5 (20.86)	**2.32 (0.52 to 4.12)**	75.5 (21.33)	0.69 (−1.10 to 2.48)
Social function	71.8 (27.55)	73.6 (27.46)	1.46 (−0.83 to 3.75)	72.7 (26.68)	0.70 (−1.58 to 2.97)

**Symptom subscales^c^**					
Fatigue	38.6 (22.60)	37.2 (22.75)	−1.18 (−3.08 to 0.73)	38.7 (22.51)	0.20 (−1.72 to 2.12)
Nausea and vomiting	5.5 (11.62)	6.0 (12.67)	0.55 (−0.59 to 1.70)	5.8 (11.78)	0.26 (−0.85 to 1.36)
Pain	31.5 (28.07)	31.8 (28.19)	0.43 (−1.89 to 2.75)	32.2 (28.33)	0.81 (−1.49 to 3.12)
Dyspnoea	20.8 (25.81)	19.0 (24.65)	−1.47 (−3.64 to 0.70)	20.11 (26.41)	−0.92 (−3.00 to 1.17)
Insomnia	41.5 (31.22)	41.5 (32.43)	0.001 (−2.81 to 2.81)	42.0 (31.15)	0.39 (−2.38 to 3.16)
Appetite loss	11.4 (21.37)	10.8 (20.83)	−0.36 (−2.28 to 1.56)	11.1 (20.65)	0.07 (−1.81 to 1.95)
Constipation	17.0 (25.79)	15.6 (24.86)	−1.51 (−3.75 to 0.73)	15.8 (25.50)	−0.68 (−2.88 to 1.52)
Diarrhoea	11.0 (20.62)	11.2 (22.66)	0.55 (−1.49 to 2.58)	12.2 (23.06)	0.89 (−1.15 to 2.92)
Financial difficulties	10.4 (23.42)	11.8 (23.82)	1.03 (−0.83 to 2.89)	11.44 (23.02)	1.02 (−0.85 to 2.89)

a *Range 0–100, higher scores reflect improved health or functioning.*

a *Bold = significant at the 5% level.*

c *Range 0–100, lower scores reflect improved symptom control. EORTC QLQ-C30 = European Organization for Research and Treatment of Cancer Core Quality of Life Questionnaire. CI = confidence interval.*

*SD = standard deviation.*

### Results for EORTC QLQ-30 subscales at 12 months

**Figure 1.**
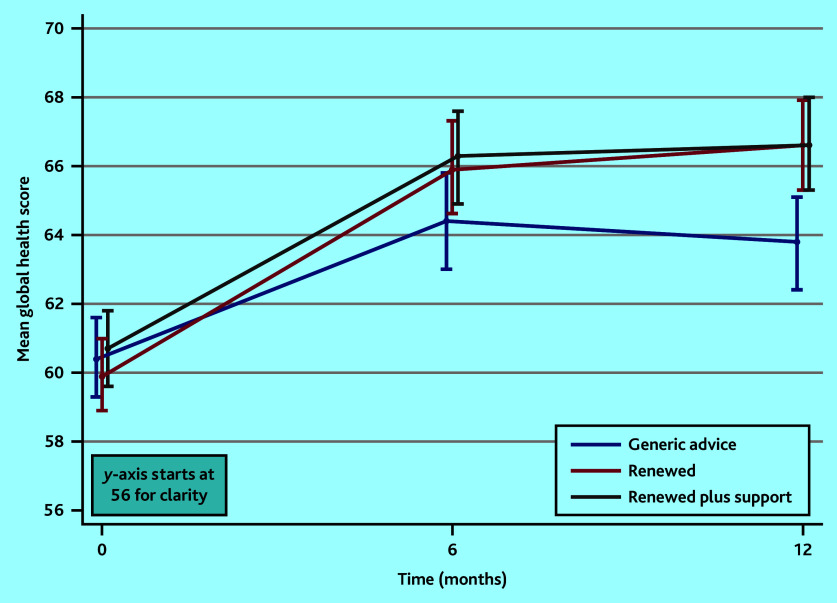
Graphical representation of improvement in the European Organization for Research and Treatment of Cancer Core Quality of Life Questionnaire global health score over time. The bars represent 95% confidence intervals.

The generic advice group did not continue to improve, and for both Renewed groups at 12 months, compared with generic advice, most subscales improved, and this was significant for global health (12 months: Renewed: 3.06, 95% CI = 1.39 to 4.74; Renewed with support: 2.78, 95% CI = 1.08 to 4.48), dyspnoea, and constipation (Table 5 and Figure 1). Enablement improved in both Renewed groups compared with generic advice (Supplementary Table S5). The Renewed with support group also showed significant improvement in the physical, emotional, cognitive, and fatigue subscales (that is, seven subscales in total).

**Table 5.** Results for EORTC QLQ-30 subscales at 12 months: means for each group, and adjusted differences between Renewed groups and generic advice

**Subscales**	**Generic advice group**	**Renewed with support**		**Renewed group**	

	**Mean (SD)**	**Mean (SD)**	**Imputed (100 imputations) (95% CI)**	**Mean (SD)**	**Imputed (100 imputations)**
**Global health^a^**	63.8 (20.46)	66.6 (19.01)	**2.78 (1.08 to 4.48)^b^**	66.6 (18.68)	**3.06 (1.39 to 4.74)**

**Functional subscales^a^**					
Physical function	78.3 (21.12)	79.6 (19.57)	**2.25 (0.88 to 3.62)**	78.5 (20.54)	0.14 (−1.22 to 1.50)
Role function	71.8 (28.63)	72.5 (27.57)	0.02 (−2.36 to 2.41)	73.2 (27.63)	0.89 (−1.50 to 3.27)
Emotional function	68.9 (23.13)	71.5 (22.35)	**2.72 (0.84 to 4.61)**	70.2 (22.82)	0.99 (−0.87 to 2.84)
Cognitive function	73.6 (22.43)	76.3 (20.59)	**1.92 (0.13 to 3.71)**	76.0 (21.33)	1.12 (−0.65 to 2.89)
Social function	72.9 (28.45)	75.6 (27.62)	2.22 (−0.20 to 4.64)	74.7 (27.83)	1.31 (−1.10 to 3.72)

**Symptom subscales^c^**					
Fatigue	38.4 (22.91)	35.6 (22.50)	**−2.67 (−4.58 to −0.75)**	37.0 (22.78)	−1.25 (−3.15 to 0.66)
Nausea and vomiting	6.1 (12.58)	5.9 (12.58)	−0.24 (−1.41 to 0.93)	5.2 (12.02)	−0.96 (−2.11 to 0.20)
Pain	31.2 (28.32)	30.9 (27.08)	−0.22 (−2.53 to 2.10)	31.8 (28.79)	0.86 (−1.48 to 3.20)
Dyspnoea	22.2 (19.26)	19.3 (25.45)	**−2.73 (−4.92 to −0.55)**	19.7 (25.31)	**−2.78 (−4.91 to −0.64)**
Insomnia	42.5 (32.56)	41.2 (32.55)	−1.30 (−4.14 to 1.55)	40.1 (30.84)	−2.27 (−5.08 to 0.54)
Appetite loss	11.0 (20.46)	10.3 (20.15)	−0.68 (−2.53 to 1.18)	10.5 (20.88)	−0.13 (−1.96 to 1.69)
Constipation	18.9 (26.29)	16.6 (25.22)	**−2.36 (−4.62 to −0.11)**	15.5 (24.95)	**−2.77 (−4.99 to −0.55)**
Diarrhoea	11.8 (21.31)	11.2 (21.60)	−0.57 (−2.51 to 1.37)	11.9 (21.40)	−0.42 (−2.38 to 1.53)
Financial difficulties	9.2 (21.60)	9.8 (21.49)	0.28 (−1.49 to 2.04)	9.5 (21.61)	0.36 (−1.40 to 2.13)

a *Range 0–100, higher scores reflect improved health or functioning.*

b *Bold = significant at the 5% level.*

c *Range 0–100, lower scores reflect improved symptom control. EORTC QLQ-C30 = European Organization for Research and Treatment of Cancer Core Quality of Life Questionnaire. CI = confidence interval.*

*SD = standard deviation.*

### Within-group analysis: improvement from baseline (post hoc analysis)

The minimally clinically important difference for the EORTC questionnaire has been determined in unwell patients with cancer for major treatments in the active phase of cancer treatment, and has been suggested as between 5 and 10.^23^^–^25 More than 40% in each of the three groups achieved a six-point improvement at 6 months with no difference between the groups, and by 12 months there was continued improvement in both the Renewed groups (Supplementary Table S4) but a flattening off for the generic advice group.

### Other secondary outcomes

As only half of the other secondary outcome questionnaire data were received, these were also presented as complete cases data. There was a modest (0.5 point) improvement in anxiety, depression, and ‘bothersomeness’ on the MYCaW1 in the Renewed with support group (Supplementary Table S5). Patient enablement improved in both the Renewed and Renewed with support groups: on average 30 out of 100 of the Renewed with support group and 35 out of 100 of the Renewed group felt enabled to manage their condition compared with those having generic advice.

#### Resource use

Mean primary care NHS costs per participant as a result of fewer appointments and prescriptions were substantially and significantly lower in the Renewed and Renewed with support groups (in comparison with generic advice £265: Renewed was –£141, 95% CI = –£153 to −£128 and Renewed with support was –£77, 95% CI = *–£*90 to −£65; see Supplementary Table S6). Although primary care was the base case analysis, since realistically the intervention was only expected to affect primary care costs, the inclusion of other community service use and/or hospital use made no difference to the inferences.

#### Results for cancer subgroups

There were no significant interaction terms and the subgroup results were generally in line with the main trial results (Supplementary Table S7). After controlling for cancer type, males did significantly better than females on the primary outcome at 6 months. There was also a suggestion that there could be more benefit among longer-term survivors.

#### Harms.

There were no reports of harms in any group. There were six deaths: one in the generic advice group, three in the Renewed with support group, and two in the Renewed group.

## Discussion

### Summary

To the authors’ knowledge, this is one of the few trials of brief multidimensional support for people with cancer and documents improvement in QoL among participants given detailed evidence-based generic lifestyle support — something that does not happen currently in everyday practice. In the shorter term (6 months) compared with generic advice there is no evidence of between-group differences in overall QoL, but global health improved in both the Renewed groups. There was a small significant difference for Renewed with support by 12 months for QoL. Modest longer-term differences for both the Renewed groups compared with generic advice were found in global rating of health, symptom management, and enablement by 12 months, with a substantial reduction in NHS primary care costs in both the Renewed groups.

### Strengths and limitations

The complex intervention was developed robustly with a user centred approach, the person-based-approach,^26^^–^28 and is one of the largest trials to assess the impact of brief support. ‘Cold calling’ invitations provide lowish uptake rates, raising concerns about generalisability. However, in the PRIMIT trial, which used similar recruitment methods,^27^ behavioural intentions were in fact lower than was subsequently found when people used the intervention outside the trial context^29^ — which suggests the authors of the current study may have underestimated the effectiveness of Renewed. Reassuringly only 24.8% (2649/10 697) declined because of internet access, and the sample was similar to observational studies of cancer survivors,^9^^,^^30^^,^^31^ which suggests generalisable results.

The choice of overall EORTC QLQ-30 score, albeit the most widely used outcome, is probably a blunt instrument for low-intensity interventions (with four-point responses for all items except global health), and other instruments might have been more suitable in retrospect — but these other instruments would have had the disadvantage of many more items for participants to complete.^32^

The ‘generic’ NHS website has lots of click-throughs to more in-depth advice and support, and there was regular follow-up by the research team that may explain the useful improvement seen in the control group. The limited number of times participants engaged is probably not a major issue: the authors of the current study have found previously with digital interventions that many people can get substantial benefit from brief engagement with core content provided it can deliver the essential advice^28^^,^^33^ and Renewed was designed with this objective — hence the importance of 96.8% of participants having used the core session.

### Comparison with existing literature

In the current study, the improvement in all groups is unlikely to reflect the natural history or regression to the mean as participants with poor QoL remain stable and consistently poor after 2 years.^5^ The primary analysis time point was chosen as 6 months because that was the timescale previous systematic reviews had reported.^34^ The small significant difference for Renewed with support by 12 months for QoL was particularly important for the subgroup with prostate cancer, helping address the need for support in this patient group.^35^^,^^36^ Compared with generic advice at 12 months for both Renewed groups there was significantly improved global health, dyspnoea, constipation, and enablement, and substantially lower NHS costs, and for Renewed with support significant differences for four other symptom subscales. This pattern makes chance a very unlikely explanation of the findings. Most important of the subscales is arguably the improvement in self-rated global health, since it has consistently been shown to be a strong predictor of mental health, physical health, and mortality in the longer term.^37^^–^43 Global health improved significantly more in both Renewed groups compared with the generic group at both 6 and 12 months, equivalent to approximately 40% of the sample rating their global health one point higher on a 13-point scale compared with the generic advice group. The finding that enablement improved with Renewed (both with and without support), albeit with less complete data, is probably also important in its own right, as in the CREW colorectal cohort study confidence to self-manage was highly predictive of subsequent health and wellbeing outcomes.^6^

There is some evidence from systematic reviews of trials that yoga, physical exercise more generally, cognitive– behavioural therapy, mindfulness-based stress-reduction programmes, and dietary interventions can improve QoL^7^^,^^34^^,^^44^^–^49 but very little evidence of benefit at 6 months from brief multidimensional home-based interventions^49^ and no evidence of benefit in the longer term. Cancer survivors are positive about digital interventions^9^ and some can be effective^9^^–^11 — albeit not sufficiently tailored, and most trials being small and few in typical primary care settings. To the authors’ knowledge there has been no trial with longer-term follow-up of robustly developed, brief multidimensional support for cancer survivors in primary care for pragmatic applicability in everyday practice.

### Implications for research and practice

Currently many cancer survivors have consistently poor QoL, but there is limited support in primary care where most participants are managed, and where resources are increasingly stretched. Cancer survivors improved with detailed online generic support, but there were further small improvements over the longer term with the bespoke Renewed intervention. The important reduction in NHS costs in primary care and benefits for symptom management and self-rated global health achieved with a very brief, scalable intervention suggests a more widespread implementation study of the Renewed intervention is warranted.

## Data

Consent was not obtained from participants for data sharing but requests for anonymised data release can be made to Paul Little (the corresponding author) with a rationale for further analyses.
